# On the Use and the Abuse of Nasal Sprays

**Published:** 1901-10

**Authors:** Dunbar Roy

**Affiliations:** Clinical Professor of Diseases of the Eye, Ear, Nose and Throat in the Atlanta College of Physicians and Surgeons, Atlanta


ATLANTA

### Journal-Record of Medicine.

Successor to Atlanta Medical and Surgical Journal, Established 1855, and Southern Medical Record, Established 1870.

VOL. III. OCTOBER, 1901. No. 7.

BERNARD WOLFF, M.D., EDITOR, Nos. 319–20 Prudential.

Published Monthly.

M. B. HUTCHINS, M.D., BUSINESS MANAGER, No. 64 Marietta St.

### *ORIGINAL COMMUNICATIONS.*

### ON THE USE AND THE ABUSE OF NASAL SPRAYS.

BY DUNBAR ROY, A.B., M.D.,

Clinical Professor of Diseases of the Eye, Ear, Nose and Throat in the Atlanta College of Physicians and Surgeons, Atlanta.

Two years ago I read a paper before the Georgia Medical Association entitled, "Some Fallacies in the Modern Treatment of Nose and Throat Diseases." In this paper I called attention to the fact that nasal sprays when rightly used were of great benefit to the patient, but when used injudiciously they were absolutely harmful. The more extended my experience becomes, the more firmly convinced am I of the truth of the above statement. Normally, the mucous membrane lining the nasal cavities and nasopharynx is sensitive in the extreme, and this lack of sensibility is present only when the membrane has undergone a chronic catarrhal condition, or when there has been a prolonged use of nasal sprays.

This first proposition is well exemplified in noting the anesthetic condition of the nasal mucous membrane as seen in patients suffering from chronic catarrhal deafness. In such, manipulations can be carried on in the nose and nasopharynx, which if attempted in others would produce the most excruciating irritation.

Overstimulation of any glands will lead to atrophy from exhaustion. One of the main functions of the nasal cavities is that of moistening and warming the inspired air before it reaches the lungs; hence, the necessity of breathing entirely through the nose, and of having a moist state of the nasal mucous membrane.

All irritation of this membrane must cause an activity of the mucous glands with consequent increased flow of the nasal secretion. This is seen, for instance, in the irritation produced by the presence of dust and chemical fumes in the atmosphere.

All substances placed in contact with the nasal cavities are irritating in various degrees—depending upon the character of the substance used and its mode of application.

Local applications are made to the nasal cavities in either of three ways: First, by means of inhalers, where the vapor is sucked into these cavities by an effort on the part of the patient; second, by sprays or atomizers, where the substance in finely divided particles is forced into the cavities by means of compressed air; third, by means of cotton on the end of a probe.

Of all these three methods the use of the spray or atomizer is possibly the most popular, but each has its sphere of usefulness.

Like all good things, the spray has been much abused.

When we speak of sprays we mean not only those used by means of compressed air, but also the various hand atomizers.

Primarily, we might say that sprays are used for three purposes: (1) for cleansing purposes; (2) for its stimulating qualities; (3) for its soothing effect upon the mucous membrane.

Secondarily, we might say that the abuse of sprays consists in: (1) the medicinal substance used; (2) the frequency of its use; (3) the force of the spray.

Let us now consider the first group.

Physiologists tell us that the mucous membrane of the two nasal cavities secrete about one pint of fluid within the twenty-fours. This, of course, is more or less evaporated in performing its physiological functions. In inflammatory conditions this amount is greatly increased, which is manifested by a discharge either anteriorly or posteriorly. In acute inflammatory conditions, as in acute coryza, there is an engorgement of the blood-vessels with transudation of serum producing a watery discharge. In chronic conditions, the submucous tissue is involved, the mucous glands are more active, and the discharge becomes decidedly thicker and mucoid in character. If there be added to this latter the presence of pus-forming organisms, the secretion may then take on a more muco-purulent character. Bacteriologists tell us that various micro-organisms normally exist in the secretions from the nasal mucous membranes.

If the mucus which is poured out from a chronically inflamed and thickened nasal membrane be allowed to remain in these cavities, such will almost certainly become semi-purulent in character, and will be a marked irritative factor in producing more secretion. This is well evidenced in children who are notoriously in the habit of not blowing their nose. For this reason a muco-purulent rhinitis in such is always difficult of cure. Children should be taught to blow out the nasal secretions at as early an age as is possible, and thus prevent much catarrhal trouble later on in life.

Nasal sprays for cleansing purposes have no place in acute inflammations, but find their chief benefits in chronic conditions where there is an abundant mucous or muco-purulent discharge. By cleansing sprays, we mean such solutions as will dissolve and cleanse off the mucus of the nasal cavities, leaving the parts in a condition to receive the proper nasal therapeusis.

The only solution which will accomplish this result is one which is watery and alkaline in character. Solutions made with Seiler's antiseptic nasal tablets, or even one with common salt and bicarbonate of soda, will accomplish this result. Of late oily sprays have become almost of universal use, but in my experience they have absolutely no place in nasal therapeusis when we desire to cleanse the nasal membrane, pure and simple.

2. Sprays used for their stimulating qualities can be either watery or oily. In these cases, what we desire to accomplish is, to stimulate and promote a healthy activity of the mucous membrane. Such a condition only arises in those chronic cases where the mucous membrane is more or less atrophic, producing a certain degree of dryness in the nasal chambers. Even in these cases there is but a limited use for the oily sprays, since the frequent application of the same has been shown to produce just this pathologic condition of the nasal mucous membrane which we are trying to obviate.

Many of the antiseptic solutions now placed upon the market contain just the ingredients necessary for a stimulating spray when properly diluted, as, for instance, listerine, 1 in 10; formalid, 1 in 8; pasturine, 1 in 8; borolyptol, 1 in 8.

For oily menstruums the following substances are useful: Camphomenthol, 20 drops to the ounce; crystal iodine, 1 grain to the ounce; terebene, 15 drops to the ounce.

3. Soothing sprays are indicated in acute congestive conditions, or where we wish to give the surfaces a soothing coating after the use of the cleansing or stimulating applications. It is here that we find the chief use of the oily spray. After a trial for several years of various substances, I find that a benzoinated oily menstruum, or one with a few drops of terebene in it, is by far the best. Benzoinated albolene is excellent. I frequently use pure white melted vaseline, using it while it is yet hot, and in the liquid state. In fact, I consider it essential for successful treatment, that all sprays of the oily variety should be heated before such enter the nasal cavities.

The abuse of sprays consists in first, the improper use of spray solutions, as has been mentioned above. The indiscriminate use of different substances in an atomizer without due reference to its suitability to the given case, is a most egregious error. Such frequently does harm to the nasal mucous membrane, and certainly does not increase the reputation of the physician. Each case is a law unto itself, and as such, requires discriminate treatment. Sprays are excellent in their place, but every case coming to the rhinologist does not require their use. In fact, many of our best rhinologists condemn the use of nasal sprays altogether, claiming that more harm than good is produced through their use. In Germany, one rarely ever sees a rhinologist make use of a spray.

If they were confined more to the pharynx and nasopharynx, there would be fewer afflicted patients.

The second abuse of sprays is the frequency of their use. This is a great and growing evil among rhinologists. The popular use of late of the menthol oily spray is largely responsible for this error. Then again, the desire on the part of the rhinologist "to be doing something" is another fruitful cause. Experience teaches me, as I have stated in a previous paper, that there exists a "spray habit," just as we find that of cocain and morphine. This occurs among patients who use the menthol spray. While menthol is a valuable nasal therapeutic remedy, I yet think that the harm it has done to nasal mucous membranes far outweighs the good. The cooling sensation produced is most refreshing to the patient, and, like cocain, seems to open up the stuffy nasal passages, but my own experience shows me that a reaction of dryness is sure to follow a prolonged use of the same, and this necessitates a more frequent application in order to overcome just this subjective sensation.

The third abuse of the spray consists in the force of the spray. This, of course, applies more particularly to those used by means of the compressed air. I have heard patients complain that when they had previously been treated, the use of the spray was always followed by severe bleeding. This I consider absolute barbarity. A compression of 15 lbs. is quite sufficient force with which to throw liquids into the nasal cavities. A force greater than 20 lbs. always causes an abrasion of the mucous membrane, especially if watery solutions be used. Traumatism of the membranes by the use of such strong compression in the sprays always works serious injury to the same. Hence judicious use in the force of a spray must always be taken into consideration.

In speaking of sprays, mention has not been made of the use of a solution of cocain. Mention is made only for the purpose of condemnation. I have never seen a case which required a spray of cocain, but I have seen cases of the cocain habit which had its origin through just such a mode of treatment on the part of the physician. I always apply cocain by means of cotton on the end of an applicator, thus limiting its action to the desired portion. If this be done, there need never be any fear of cocain intoxication. I think that it is almost criminal to spray a solution of cocain in the nose, especially if it be a chronic case, for I am firmly convinced that the number of cocain habitués would be lessened if this advice was heeded.

In conclusion, allow me to say that the views herein expressed are those deduced from my own personal experience and in no way authoritative. Each rhinologist naturally thinks for himself, and I doubt not but that there are many such who will not agree with these ideas of mine, but if I can cause nose- and throat-workers to study more thoroughly the use of nasal sprays, then I shall feel as if this paper has accomplished some good.

*Grand Opera House.*

